# Neurodegeneration: Etiologies and New Therapies 2016

**DOI:** 10.1155/2016/8363179

**Published:** 2016-11-03

**Authors:** Eng King Tan, Amit K. Srivastava, W. David Arnold, Mahendra P. Singh, Yiying Zhang

**Affiliations:** ^1^Department of Neurology, Singapore General Hospital, National Neuroscience Institute and Duke NUS Graduate Medical School, Singapore 169857; ^2^Department of Pediatric Surgery, University of Texas Health Sciences Center at Houston, Houston, TX 77030, USA; ^3^Department of Neurology, Division of Neuromuscular Disorders and Department of PM&R and Department of Neuroscience, The Ohio State University Wexner Medical Center, Columbus, OH 43210, USA; ^4^Toxicogenomics and Predictive Toxicology Division, CSIR-Indian Institute of Toxicology Research, Lucknow, Uttar Pradesh 226 001, India; ^5^Department of Anesthesia, Critical Care and Pain Medicine, Massachusetts General Hospital, Harvard Medical School, Charlestown, MA 02129, USA

In this special issue, we have 9 articles that highlight diverse biochemical, immunological, molecular, and neuroimaging techniques used to decipher the pathophysiological mechanisms underpinning neurodegeneration in various cellular and animal models. The common diseases are covered, including a review on the therapeutic options in Alzheimer's disease and a discussion on the factors influencing homocysteine levels in Parkinson's disease. Of particular interest in this issue is an article on the entity of “chemo brain,” a condition that overlaps between oncology and neurology. “Chemo brain” is a common term used to describe thinking and memory problems that can occur after cancer treatment. Here the authors demonstrate PET evidence of the effect of donepezil on cognitive performance in an animal model. We hope this special issue will generate further interest and debate on the pathoetiology of neurodegenerative diseases and provide a platform to generate impetus to further identify and validate new therapeutic options.

*Eng King Tan* *Amit K. Srivastava* *W. David Arnold* *Mahendra P. Singh* *Yiying Zhang*

